# Distinct Roles of Perilipins in the Intramuscular Deposition of Lipids in Glutamine-Supplemented, Low-, and Normal-Birth-Weight Piglets

**DOI:** 10.3389/fvets.2021.633898

**Published:** 2021-06-21

**Authors:** Yaolu Zhao, Elke Albrecht, Zeyang Li, Johannes Schregel, Miriama Sciascia, Cornelia C. Metges, Steffen Maak

**Affiliations:** ^1^Institute of Muscle Biology and Growth, Leibniz Institute for Farm Animal Biology (FBN), Dummerstorf, Germany; ^2^Institute of Nutritional Physiology “Oskar Kellner”, Leibniz Institute for Farm Animal Biology (FBN), Dummerstorf, Germany

**Keywords:** glutamine, low birth weight, lipid deposition, muscle gene expression, perilipins, pig

## Abstract

Piglets with low birth weight (LBW) usually have reduced muscle mass and increased lipid deposition compared with their normal-birth-weight (NBW) littermates. Supplementation of piglets with amino acids during the first days of life may improve muscle growth and simultaneously alter the intramuscular lipid deposition. The aim of the current study was to investigate the influence of glutamine (Gln) supplementation during the early suckling period on lipid deposition in the longissimus muscle (MLD) and the role of different perilipin (PLIN) family members in this process. Four groups were generated consisting of 72 male LBW piglets and 72 NBW littermates. Piglets were supplemented with either 1 g Gln/kg body weight or an isonitrogenous amount of alanine (Ala) between days post natum (dpn) 1 and 12. Twelve piglets per group were slaughtered at 5, 12, and 26 dpn, and muscle tissue was collected. Perilipins were localized by immunohistochemistry in muscle sections. The mRNA and protein abundances of PLIN family members and related lipases were quantified by quantitative RT-PCR (qPCR) and western blots, respectively. While PLIN1 was localized around lipid droplets in mature and developing adipocytes, PLIN2 was localized at intramyocellular lipid droplets, PLIN3 and 4 at cell membranes of muscle fibers and adipocytes, and PLIN5 in the cytoplasm of undefined cells. The western blot results indicated higher protein abundances of PLIN2, 3, 4, and 5 in LBW piglets (*p* < 0.05) at 5 dpn compared with their NBW littermates independent of supplementation, while not directly reflecting the mRNA expression levels. The mRNA abundance of *PLIN2* was lower while *PLIN4* was higher in piglets at 26 dpn in comparison with piglets at 5 dpn (*p* < 0.01). Relative mRNA expression of *LPL* and *CGI-58* was lowest in piglets at 5 dpn (*p* < 0.001). However, *ATGL* mRNA was not influenced by birth weight or supplementation, but the Spearman correlation coefficient analysis revealed close correlations with *PLIN2*, 4, and 5 mRNA at 5 and 26 dpn (*r* > 0.5, *p* < 0.001). The results indicated the importance of birth weight and age for intramuscular lipid deposition and different roles of PLIN family members in this process, but no clear modulating effect of Gln supplementation.

## Introduction

Low-birth-weight (LBW) piglets normally have a higher mortality rate and growth retardation of muscles compared with their normal-birth-weight (NBW) littermates, which in turn causes a delay in whole body growth (1). Furthermore, LBW piglets normally have greater fat deposition (2) and exhibit increased fatness at slaughter age (1). In the current project, we attempted to ameliorate the disadvantages of the LBW piglets with adapted nutrition, in particular with glutamine (Gln) supplementation, which was assumed insufficient in maternal milk especially for LBW piglets (3, 4). In our previous investigation (5), we observed fewer muscle fibers and more intramyocellular lipid droplets at 5 days post natum (dpn) in LBW piglets. Moreover, Gln supplementation was shown to increase intramuscular availability of free Gln in skeletal muscle in a short term and to influence muscle fiber size, but had no influence on further muscle morphology traits and a minor influence on the abundance of myosin heavy chain isoforms. The current study focused on the process of lipid deposition and redistribution between muscle fibers and developing adipocytes as part of the muscle development and how it was influenced by birth weight (BiW) and Gln supplementation.

Skeletal muscle development includes myogenesis, adipogenesis, as well as fibrogenesis (6). During the whole lifetime of pigs, lipogenesis interplays and competes with muscle growth (7, 8). However, the lipid content in skeletal muscle is not just a storage of nutritional energy; it is of great importance for meat quality (9). Appropriate intramuscular lipids in pork promote flavor, tenderness, and juiciness (10), and consequently, intramuscular lipid development has become increasingly importantly raised in pork production. Intramuscular fat in pig represents only a small part of total body fat (6). The lipids within skeletal muscle are either stored in intramuscular adipocytes or within muscle fibers as intramyocellular lipid droplets (5). These intramuscular lipid droplets, also named lipid or fat bodies, play a vital role in lipid homeostasis in skeletal muscle (11). They function in storing lipids and supplying energy (11–13), as well as providing lipids for membrane synthesis (14) and protecting the cells from lipotoxicity (15). Moreover, the intramuscular lipid droplets were found to be formed *de novo* (16), and their formation and physical functions are controlled by a series of proteins, such as synaptosomal-associated protein 23 (SNAP23) (17), caveolins (18), PAT family (19), and others. Among these proteins, PAT family proteins, named after perilipin, adipophilin, or adipose differentiation-related protein (ADRP), and the tail-interacting protein of 47 kDa (TIP47) (20) have drawn increasing attention in recent years. There are two new members in this family, S3–12 (21) and myocardial lipid droplet protein—MLDP (22), also known as OXPAT (23) or lipid storage droplet protein 5 (LSDP5) (24). These five proteins, also known as PLIN1–5 (25), participate in the process of lipid droplet formation, stabilization, and lipolysis in different cells (26). The encoding genes can serve as suitable biomarkers when studying lipid deposition and adipocyte development within skeletal muscle in piglets.

Our hypothesis was that differences in intramuscular lipid deposition between LBW and NBW piglets are modulated by Gln supplementation, and different PLIN family members are involved in this process. Therefore, the objective of the current study was to investigate the expression pattern of *PLINs* in LBW and NBW piglets during the first weeks after birth on mRNA and protein level and their modulation by Gln supplementation.

## Materials and Methods

### Animals and Sampling

The study involved German Landrace male piglets with 72 LBW and 72 NBW littermates, nursed and supplemented with Gln or an isonitrogenous amount of alanine (Ala) as described in detail in our former publication (5). Twelve piglets per group were sacrificed at 5, 12, or 26 dpn. The four treatment groups were named LBW-GLN, LBW-ALA, NBW-GLN, and NBW-ALA. Experimental procedures and animal care were carried out strictly according to the European Convention for the Protection of Vertebrate Animals used for Experimental and Other Scientific Purposes (2010/63/EU) and were approved by the responsible State Office for Agriculture, Food Safety and Fishing Mecklenburg Western Pomerania, Germany (permission no. 7221.3-1-026/16). Tissue of *musculus longissimus dorsi* (MLD) and *musculus semitendinosus* (MST) was collected immediately after slaughter, snap frozen in liquid nitrogen, and subsequently stored at −80°C until analysis.

### Muscle Histology

Muscle serial sections of MLD and MST were cut 10-μm thick using a cryostat microtome (CM3050 S, Leica, Bensheim, Germany). The muscle sections were stained with hematoxylin and eosin (H/E, hematoxylin: Dako, Glostrup, Denmark; eosin: Chroma Gesellschaft, Münster, Germany) or Oil Red O according to standard protocols. The H/E-stained images were used for measurement, and the Oil Red O-stained images were used to verify the selected adipocytes. Images were taken with an Olympus BX43 microscope (Olympus, Hamburg, Germany) equipped with a UC30 color camera and analyzed with Cell^∧^D imaging software (OSIS, Münster, Germany). At least 200 intramuscular adipocytes in both muscles were measured for each piglet using the interactive measurement module of the Cell^∧^D software. If <200 adipocytes could be found, as in many of 5 dpn piglets, all available adipocytes were measured.

### RNA Isolation, cDNA Synthesis, and Quantitative RT-PCR

Muscle RNA was isolated from MLD pieces (70–90 mg) using the RNeasy Fibrous Tissue Mini Kit (Qiagen, Hilden, Germany), following the manufacturer's instructions, and stored at −80°C. All RNA concentrations were measured with a NanoDrop ND-1000 spectrophotometer (Peqlab, Erlangen, Germany), and RNA integrity was determined using the Experion Automated Electrophoresis System and the RNA StdSens analysis chip (Bio-Rad, Munich, Germany). Then, first-strand cDNA was synthesized in a 20-μl reaction volume from 150 ng RNA with an iScript cDNA Synthesis Kit (Bio-Rad). Primers of reference and target genes were designed with Primer 3 web version 4.1.0 (http://primer3.ut.ee/) or adapted from published papers [tyrosine 3-monooxygenase/tryptophan 5-monooxygenase activation protein zeta, YWHAZ (27); peptidylprolyl isomerase A, PPIA (28)], as shown in Table 1. All primers were synthesized by a commercial company (Sigma-Aldrich, Darmstadt, Germany). The annealing temperature of all primers was 60°C. To test these primers, qualitative polymerase chain reaction (PCR) was performed, and the products were subjected to 3% agarose gel electrophoresis and sequenced as described by Liu et al. (29). The quantitative RT-PCR (qPCR) was performed in duplicate as described by Schering et al. (30) with FastStart Essential DNA Green Master using a LightCycler^®^ 96 real-time qPCR system (Roche, Basel, Switzerland). The mRNA expression values of target genes were normalized to two stable reference genes: *YWHAZ* and *PPIA*. The quantitation cycle (Cq) value was analyzed by the LightCycler^®^ 96 system software. Efficiencies of amplifications were calculated by standard curves, which were calculated from serial dilutions (1:1, 1:10, 1:50, 1:100, and 1:200) using the formula $\left(\;Efficiency=10^{-\frac{1}{slope}}\right)$ and were within 1.8–2.2. The mRNA abundances were calculated as normalized relative quantities (NRQ) (31).

**Table 1:** Primer sequences used for qPCR.

**Gene**	**Accession number**	**Forward primer (5′)**	**Reverse primer (3′)**	**Product size, bp**
*YWHAZ*	NM_001315726.1	ATGCAACCAACACATCCTATC	GCATTATTAGCGTGCTGTCTT	178
*PPIA*	NM_214353.1	CACAAACGGTTCCCAGTTTT	TGTCCACAGTCAGCAATGGT	171
*PLIN1*	NM_001038638.1	GGGGTGTTGAGAAGGTGGTA	GGTGTGTTGAGAGATGGTGC	155
*PLIN2*	NM_214200.2	ATTGCCAACACTTACGCCTG	CGGTCACTGCTTCTTTGGTC	208
*PLIN3*	NM_001031778.1	ATCAGAGCTACTTCGTGCGT	AGTTTCTCCTGACCCTCCAC	196
*PLIN4*	NC_010444.4	CTGAGCAGCTTCTTTGGGTC	GGCTCCAGAGATCACCTTGT	219
*PLIN5*	NM_001123135.1	CCCTTTCTTCAGCAGCCTTC	GAGCTCCTCCTCAGTCATGG	207
*LPL*	NM_214286.1	CAGAGCCAAAAGAAGCAGCA	GGATGTTTTCACTCTCGGCC	170
*CGI-58*	NM_001012407.1	TCCCCTTGTCCTCCTTCATG	GGTTGTGTCCCAGCAAGATC	233
*ATGL*	NM_001098605.1	AGCACCTTCATTCCCGTGTA	TGGATGTTGGTGGAGCTGTC	176

### Immunohistochemistry

Tissue sections of MLD (thickness 8 μm) were cut with a CM3050 S cryostat microtome (Leica, Bensheim, Germany) and stained with antibodies against PLIN1–5 that were purchased from Novus Biologicals (NB110-40760, NB110-40878, NB110-40765, NBP2-13776, and NB110-60509; Wiesbaden-Nordenstadt, Germany). Fixation of muscle sections was done for 15 min in 4% paraformaldehyde. After washing 3 × 5 min with phosphate-buffered saline (PBS), the slides were incubated with 10% normal goat serum (NGS) in PBS for 15 min to block non-specific binding of the secondary antibody. Primary antibody (dilution 1:200 for PLIN1 and 4 and 1:100 for PLIN2, 3, and 5) incubation was performed for 1 h. Then, the slides were rinsed briefly, washed 3 × 10 min with PBS and subsequently incubated in the dark with secondary antibody (Alexa Fluor 488 Goat-Anti-Rabbit IgG, 1:1,000; Life Technologies, Darmstadt, Germany) for 45 min. Slides were washed 3 × 10 min with PBS, and the nuclei were stained with Hoechst 33258 for 5 min. After being washed 2 × with PBS and 1 × with distilled water for 5 min each, the slides were covered with ProLong Antifade (Thermo Fisher Scientific, Schwerte, Germany). All incubations were performed at room temperature in a humidity chamber. Two types of negative controls were generated to detect non-specific antibody binding, either blocking the primary antibodies with respective blocking peptides or omitting the primary antibody. No non-specific secondary antibody binding was detected, but weak non-specific binding of the antibodies against PLIN1 and 5 was observed (Supplementary Figure 1). Fluorescence signals were observed with a Nikon Microphot SA fluorescence microscope (Nikon, Düsseldorf, Germany) and a CC-12 color camera and recorded with Cell^∧^F image analysis software (OSIS, Münster, Germany).

### Western Blotting

Protein was isolated from all 144 MLD samples as described in details by Liu et al. (29) using CelLytic MT lyses reagent (Sigma-Aldrich, Munich, Germany) and protease inhibitor. Protein mixed with loading buffer was denatured at 95°C for 5 min and then loaded on Criterion TGX 12% gels (Bio-Rad) together with a molecular weight marker (PageRuler, Thermo Scientific, Schwerte, Germany). After electrophoresis, proteins were transferred to a polyvinylidene difluoride (PVDF) membrane (Trans-Blot Turbo Transfer Pack, Bio-Rad) with a semi dry blotter (Trans-Blot, Bio-Rad). The Smart Protein Layers (SPL) Western Kit Red (PR913-R, NH DyeAGNOSTICS, Halle, Germany) was used to enable reliable protein quantification. Membranes were blocked with 5% non-fat dry milk or 10% ROTI Block (Carl Roth, Karlsruhe, Germany) in Tris-buffered saline (TBS) for 1 h at room temperature. All membranes were incubated with the primary antibodies, which were also used for immunohistochemistry (PLIN1 and 2 diluted at 1:15,000 and PLIN3, 4, and 5 diluted at 1:5,000) and a HRP-conjugated secondary antibody (Rabbit TrueBlot HRP, Rockland Immunochemicals, Limerick, PA, USA; diluted at 1:25,000). The antibody label was detected with highly sensitive chemiluminescence substrate (SuperSignal West Femto, Thermo Scientific). Chemiluminescence and fluorescence of calibrators and total protein were recorded with a ChemoCam HR-16 imager (INTAS, Göttingen, Germany) and quantified with LabImage 1D software (Kapelan Bio-Imaging, Leipzig, Germany). Target protein band volume was normalized to total protein abundance, and the SPL method enabled comparability among blots. All 144 samples were measured at least twice on separate blots (see example full blots in Supplementary Figure 2). Negative controls were generated to discriminate specific and non-specific antibody binding. Separate blots were processed that were incubated with the antibodies alone or with the antibodies together with respective blocking peptides, pre-incubated for 30 min (Supplementary Figure 3).

### Statistical Analysis

Statistical analysis was performed with the analysis of variance (ANOVA) model using the MIXED procedure of SAS statistical software (version 9.4, SAS Inst., Cary, USA). The fixed factors were BiW (LBW or NBW), supplementation (ALA or GLN), and age (5, 12, or 26), and respective interactions and sow were included as random factors. The used model was as follows:

*Y*_*ijksl*_ = μ + BiW_*i*_ + Sup_*j*_ + Age_*k*_ + (BiW × Sup)_*ij*_ + (BiW × Age)_*ik*_ + (Sup × Age)_*jk*_ + (BiW × Sup × Age)_*ijk*_ + Sow_*s*_ + *e*_*ijksl*_,

where *Y*_ijksl_ = dependent variable (animal *l* from Sow *s* in BiW group *i*, Sup group *j*, and Age group *k*); μ = overall mean; BiW_*i*_ = effect of birth weight *i* (*i* = 1–2); Sup_j_ = effect of supplementation *j* (*j* = 1–2); Age_*k*_ = effect of age *k* (*k* = 1–3); (BiW × Sup)_*ij*_ = effect of interaction; (BiW × Age)_*ik*_ = effect of interaction; (Sup × Age)_*jk*_ = effect of interaction; (BiW × Sup × Age)_*ijk*_ = effect of interaction; Sow_*s*_ = random effect of sow *s* (*s* = 49); and *e*_*ijksl*_ = random residual error.

Least-square means (LSmeans) and standard errors (SE) were calculated for each fixed effect, and pairwise differences were tested by the Tukey–Kramer test. The SLICE statement of the MIXED procedure was used for partitioned analysis of the LSmeans for the interaction between BiW and supplementation within ages. Differences were considered significant if Tukey–Kramer adjusted *p* < 0.05 and a trend if 0.1 > *p* ≥ 0.05. The CORR procedure of SAS was used to calculate Spearman correlation coefficients. Correlations were regarded as low (0.3 < *r* < 0.5), moderate (0.5 < *r* < 0.7), high (0.7 < *r* < 0.9), and very high (*r* > 0.9) as described by Mukaka et al. (32).

## Results

### Lipid Droplets and Adipocyte Size in *M. longissimus* and *M. semitendinosus*

**Figure 1:**
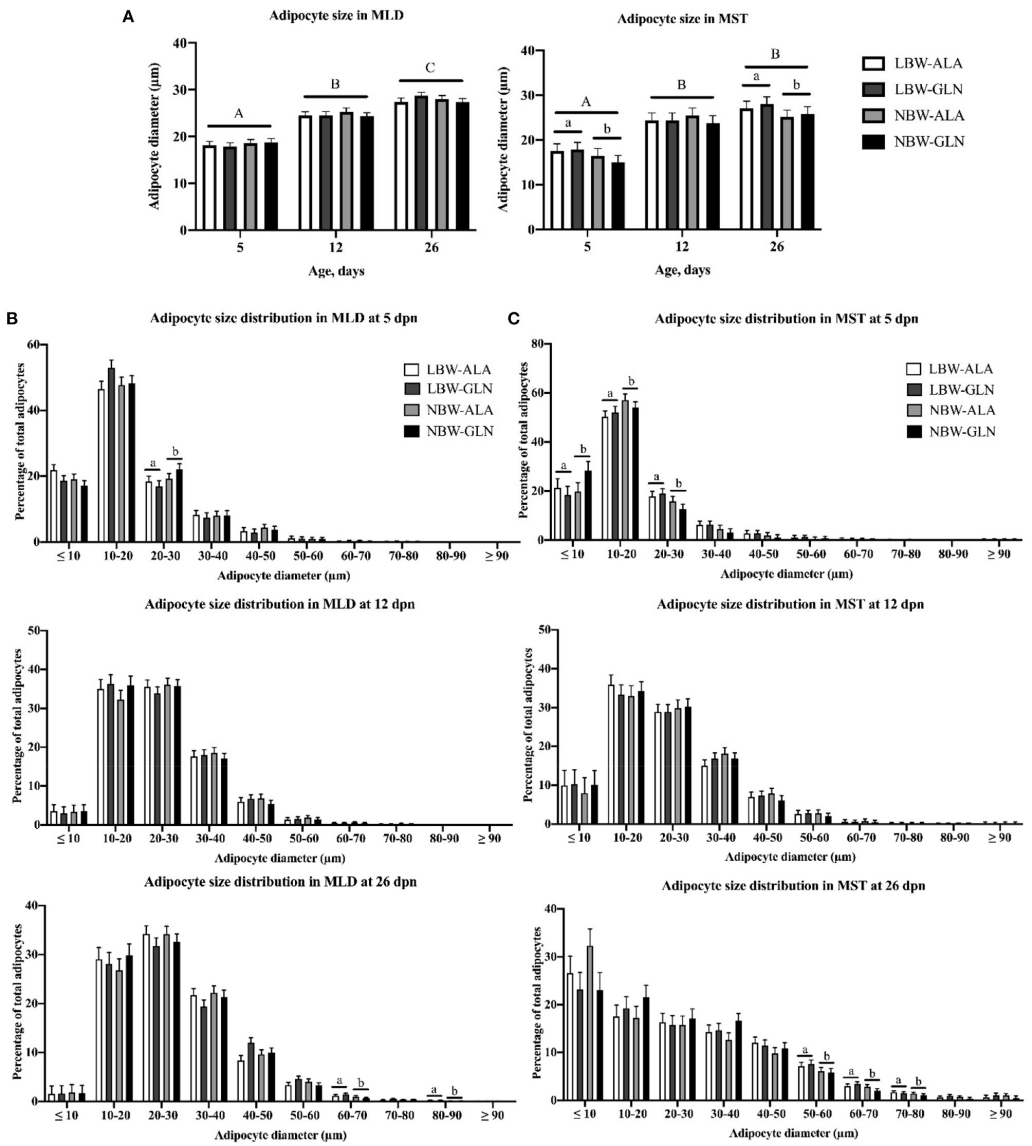
Intramuscular adipocyte diameter and adipocyte size frequency distribution within *M. longissimus* (MLD) and *M. semitendinosus* (MST) of low- (LBW) and normal-birth-weight (NBW) piglets at 5, 12, and 26 dpn, supplemented with glutamine (GLN) or alanine (ALA) (*n* = 12 per group). **(A)** Intramuscular adipocyte diameter within MLD and MST; intramuscular adipocyte size distribution within **(B)** MLD and **(C)** MST. Values represent LSmeans and SE in error bars. ^a,b^Significant differences between LBW and NBW piglets; ^A−*C*^Significant differences among ages (*p* < 0.05, Tukey–Kramer test).

Results of total lipid droplet area in muscle fibers and in adipocytes were reported in our former publication (5). We observed more lipid droplets in LBW piglets than in their NBW littermates at 5 dpn. Differences disappeared in older animals, and an influence of Gln supplementation was not detected. In the current study, we measured the size of individual intramuscular adipocytes. Adipocyte size distributions in MLD and MST are presented in Figure 1. The adipocyte size was mainly influenced by age and BiW, but no significant interaction effect among BiW, supplementation, and age was observed (*p* > 0.05). In both muscles, the mean adipocyte diameter was larger in piglets at 12 (*p* ≤ 0.001) or 26 dpn (*p* < 0.001) compared with animals at 5 dpn (Figure 1A). Size histograms showed that piglets at 26 dpn had more large adipocytes than at 5 or 12 dpn (Figures 1B,C). Comparing the adipocyte size distribution of MLD revealed more small-diameter adipocytes (20–30 μm, *p* = 0.03) at 5 dpn in NBW piglets and more large-diameter adipocytes (60–70 μm, *p* = 0.016 and 80–90 μm, *p* < 0.001) at 26 dpn in LBW piglets (Figure 1B). In addition, we found more medium-diameter fat cells (40–50 μm) in Gln-supplemented piglets at 26 dpn than in Ala-supplemented animals, independent of BiW groups. In MST, the mean diameter of adipocytes of LBW piglets was greater than that of their NBW littermates at 5 (*p* = 0.038) and 26 dpn (*p* = 0.03), which was not observed in MLD. Furthermore, NBW piglets had more small-size adipocytes (<20 μm, *p* < 0.05), whereas LBW animals had more medium-size adipocytes (20–30 μm, *p* = 0.012 and 30–40 μm, *p* = 0.077) at 5 dpn. At 26 dpn, LBW piglets had more large-diameter fat cells (50–80 μm, *p* < 0.05) in MST than NBW piglets. Fewer adipocytes with a diameter smaller than 10 μm were observed in GLN-supplemented piglets in comparison with Ala-supplemented animals (*p* = 0.037), regardless of BiW group.

### Relative mRNA Abundance of PLIN Family Members and Interacting Lipolysis-Related Genes in *M. longissimus*

**Figure 2:**
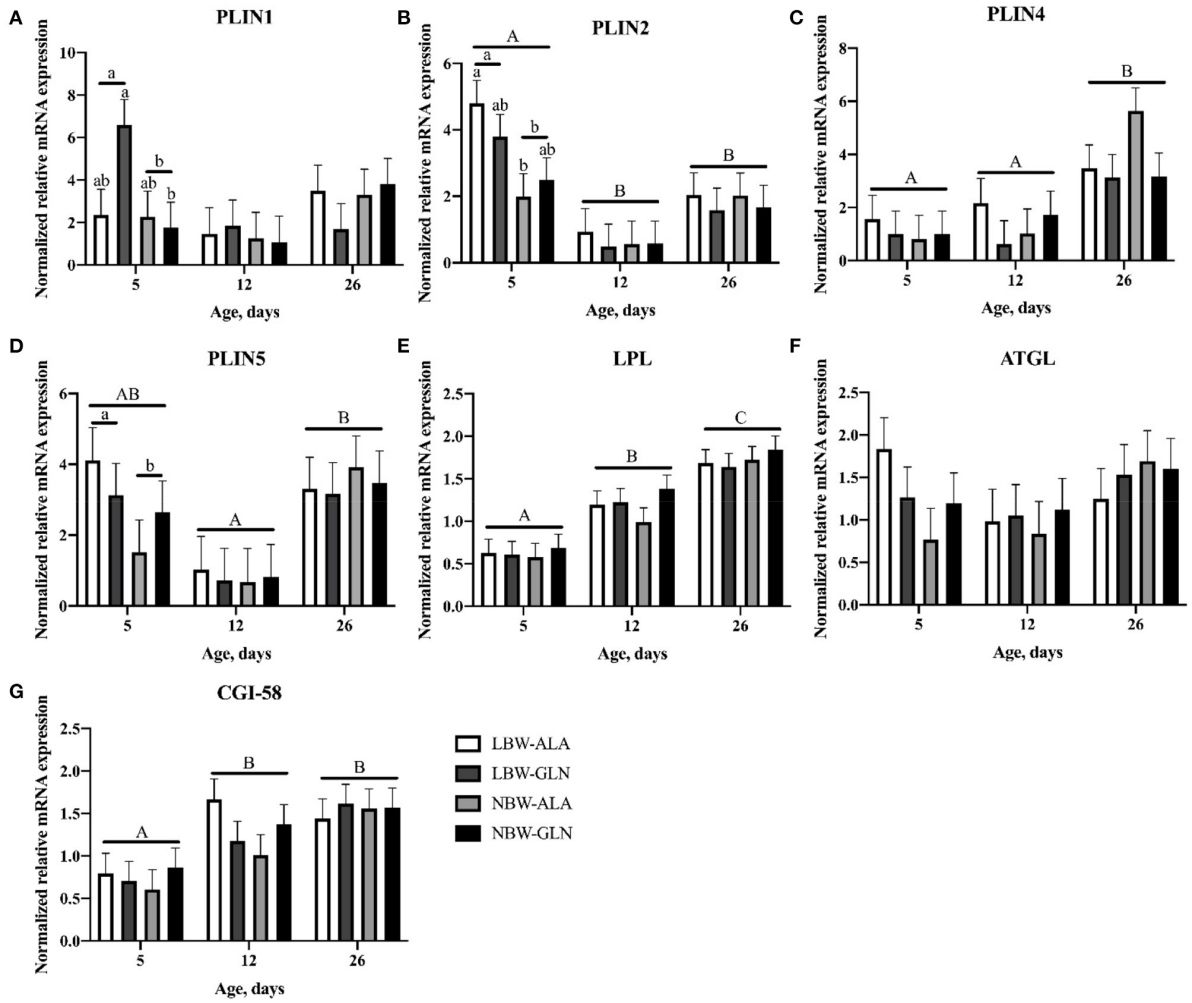
Normalized relative mRNA expression of perilipins and related lipases in *M. longissimus* of low- (LBW) and normal-birth-weight (NBW) piglets at 5, 12, and 26 dpn, supplemented with glutamine (GLN) or alanine (ALA) (*n* = 12 per group). Values represent LSmeans and SE in error bars of PLIN1 **(A)**, PLIN2 **(B)**, PLIN4 **(C)**, PLIN5 **(D)**, LPL **(E)**, ATGL **(F)**, and CGI-58 **(G)** mRNA abundance, normalized to YWHAZ and PPIA. ^a,b^Significant differences between LBW and NBW piglets at the same age; ^A−*C*^Significant differences among ages (*p* < 0.05, Tukey–Kramer test).

The abundance of *PLIN1–5* and interacting lipolysis-related genes was quantified with RT-qPCR (Figure 2) to elucidate their contribution to the observed differences in lipid deposition between LBW and NBW piglets. The statistical analyses revealed no interaction effect among BiW, supplementation, and age for the mRNA abundances of the investigated genes (*p* > 0.05). Age and BiW were also the main influencing factors for the mRNA abundances of the investigated genes. The *PLIN1* mRNA level was overall higher in LBW than in NBW piglets at 5 dpn (*p* = 0.033), and in particular, it was higher in LBW-GLN piglets than in NBW-GLN piglets (*p* = 0.017, Figure 2A). However, no significant effects of supplementation (*p* = 0.53) or age (*p* = 0.127) were observed. Relative mRNA expression of *PLIN2* was higher in piglets at 5 dpn in comparison with animals at 12 (*p* < 0.001) or 26 dpn (*p* = 0.04, Figure 2B). Furthermore, LBW-ALA piglets had more *PLIN2* mRNA compared with their NBW-ALA littermates at 5 dpn (*p* = 0.008). Independent of supplementation, *PLIN2* mRNA abundance was greater in LBW piglets than in NBW piglets at 5 dpn (*p* = 0.001). However, this effect was not observed at 12 (*p* = 0.811) or 26 dpn (*p* = 0. 949), and no supplementation effects were detected (*p* = 0.462) independent of ages. The relative mRNA abundance of *PLIN3* was too low to be reliably detected in MLD of piglets in this study. The abundance of *PLIN4* mRNA was lower in piglets at 5 dpn (*p* = 0.005, Figure 2C) and 12 dpn (*p* = 0.014) compared with piglets at 26 dpn and was not influenced by BiW (*p* = 0.570) or supplementation (*p* = 0.172). The abundance of *PLIN5* mRNA in piglets at 12 dpn tended to be lower than in piglets at 5 dpn (*p* = 0.062, Figure 2D) and was lower than in animals at 26 dpn (*p* < 0.001). Furthermore, LBW-ALA piglets tended to have higher *PLIN5* mRNA levels than NBW-ALA piglets at 5 dpn (*p* = 0.074). Overall, independent of Gln supplementation, LBW piglets had more *PLIN5* mRNA compared with NBW at 5 dpn (*p* = 0.042), but not at 12 (*p* = 0.874) or 26 dpn (*p* = 0.538). The results did not show an effect of Gln supplementation on *PLIN5* mRNA abundance (*p* = 0.851).

Birth weight and Gln supplementation had no effect on mRNA abundance of *LPL* (*p* = 0.641 and *p* = 0.29, respectively). There was only an age effect detected; thus, more *LPL* mRNA was measured in piglets at 26 dpn compared with piglets at 5 (*p* < 0.001, Figure 2E) and 12 dpn (*p* < 0.001). Similarly, the mRNA level of *CGI-58* was higher in piglets at 12 (*p* = 0.026, Figure 2F) and 26 dpn (*p* < 0.001) compared with animals at 5 dpn. No BiW (*p* = 0.561) or Gln supplementation (*p* = 0.774) effects were observed. There was a trend for higher level of *ATGL* mRNA in LBW-ALA compared with NBW-ALA piglets at 5 dpn (*p* = 0.054). Moreover, *ATGL* mRNA tended to be higher in LBW piglets than in NBW animals at 5 dpn independent of supplementation (*p* = 0.054), but it was not affected by Gln supplementation (*p* = 0.738) or age (*p* = 0.335).

### Localization of PLIN Proteins Within *M. longissimus*

**Figure 3:**
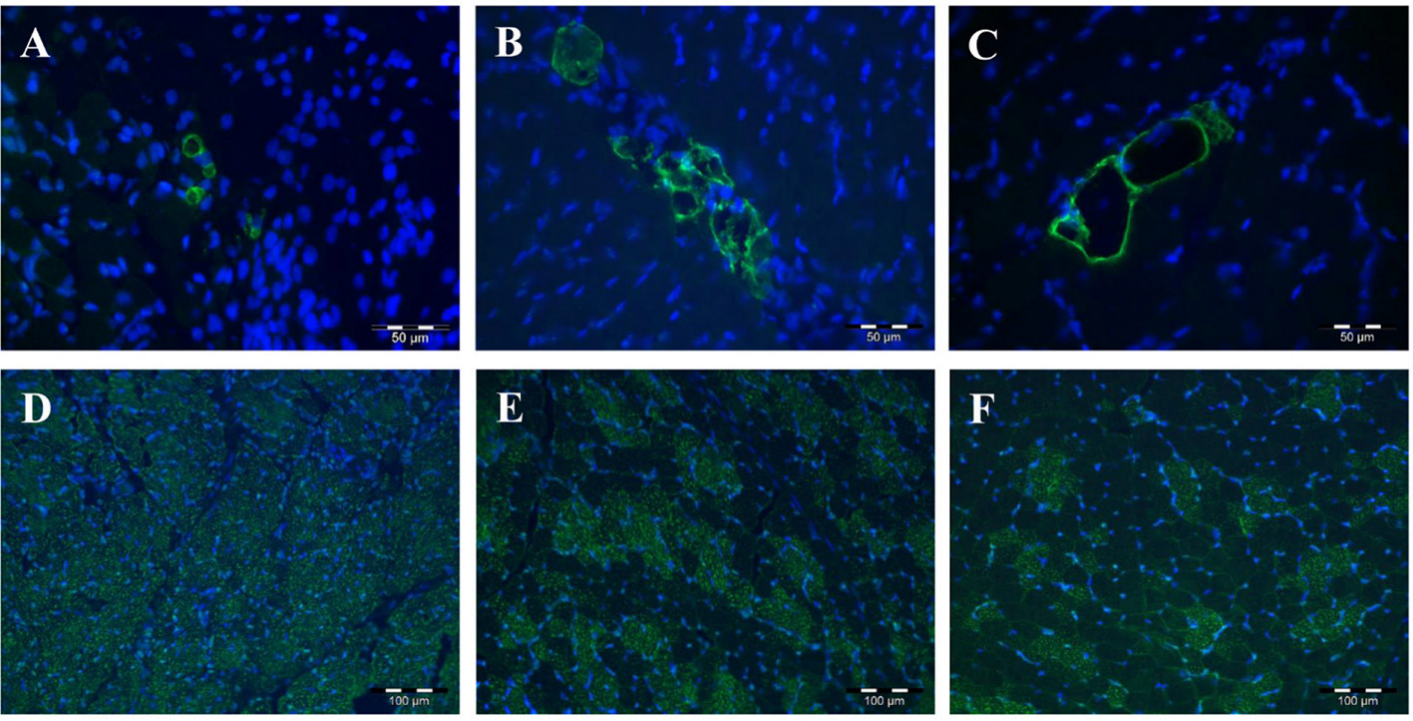
Immunohistochemical localization of PLIN1 **(A–C)** and PLIN2 **(D–F)** in porcine *M. longissimus* at 5, 12, and 26 dpn, respectively. PLIN1 and 2 were stained green. Nuclei were counterstained blue with Hoechst 33258.

**Figure 4:**
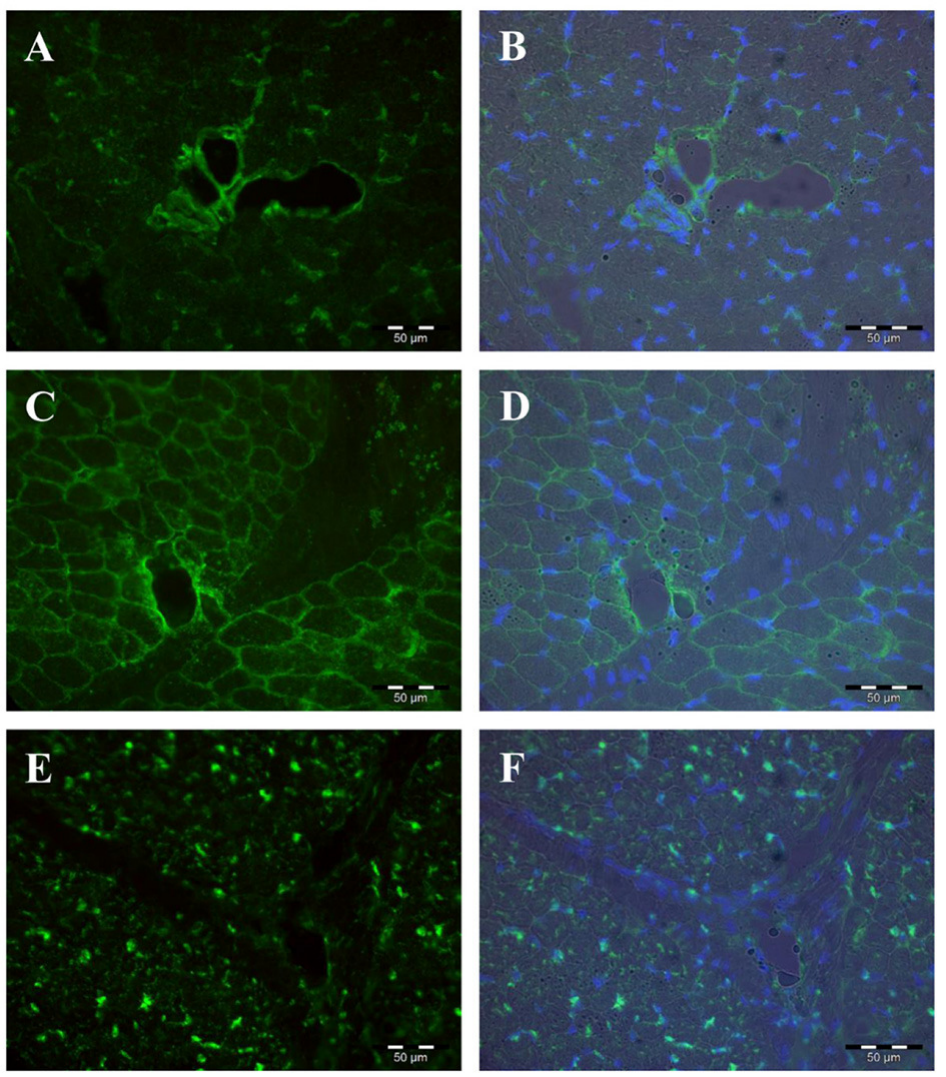
Immunohistochemical localization of PLIN3 **(A,B)**, PLIN4 **(C,D)**, and PLIN5 **(E,F)** in porcine *M. longissimus* at 12 dpn. Nuclei were counterstained blue with Hoechst 33258. The right panel shows merged images of fluorescence (PLIN and Hoechst 33258) and bright field.

The proteins encoded by the five *PLIN* genes were detected with immunohistochemistry in the MLD of piglets. Different localizations and staining patterns were observed for the individual PLIN proteins. A strong signal was observed for PLIN1 around fat vacuoles in mature and developing adipocytes (Figures 3A–C). Additional weak signals in muscle tissue were identified as unspecific (see Supplementary Figure 1) by blocking specific antibody bindings with the respective blocking peptide. In contrast, PLIN2 was localized exclusively at intramyocelluar lipid droplets (Figures 3D–F). The staining decreased with age according to the reduction of lipid droplets within muscle fibers. Similar staining patterns were observed for PLIN3 and PLIN4, both located at the periphery of muscle fibers and large adipocytes (Figures 4A–D), whereas PLIN5 was detected in the cytosol of many small, undefined cells between muscle fibers (Figures 4E,F).

### Protein Abundance of PLIN1–5 in *M. longissimus*

**Figure 5:**
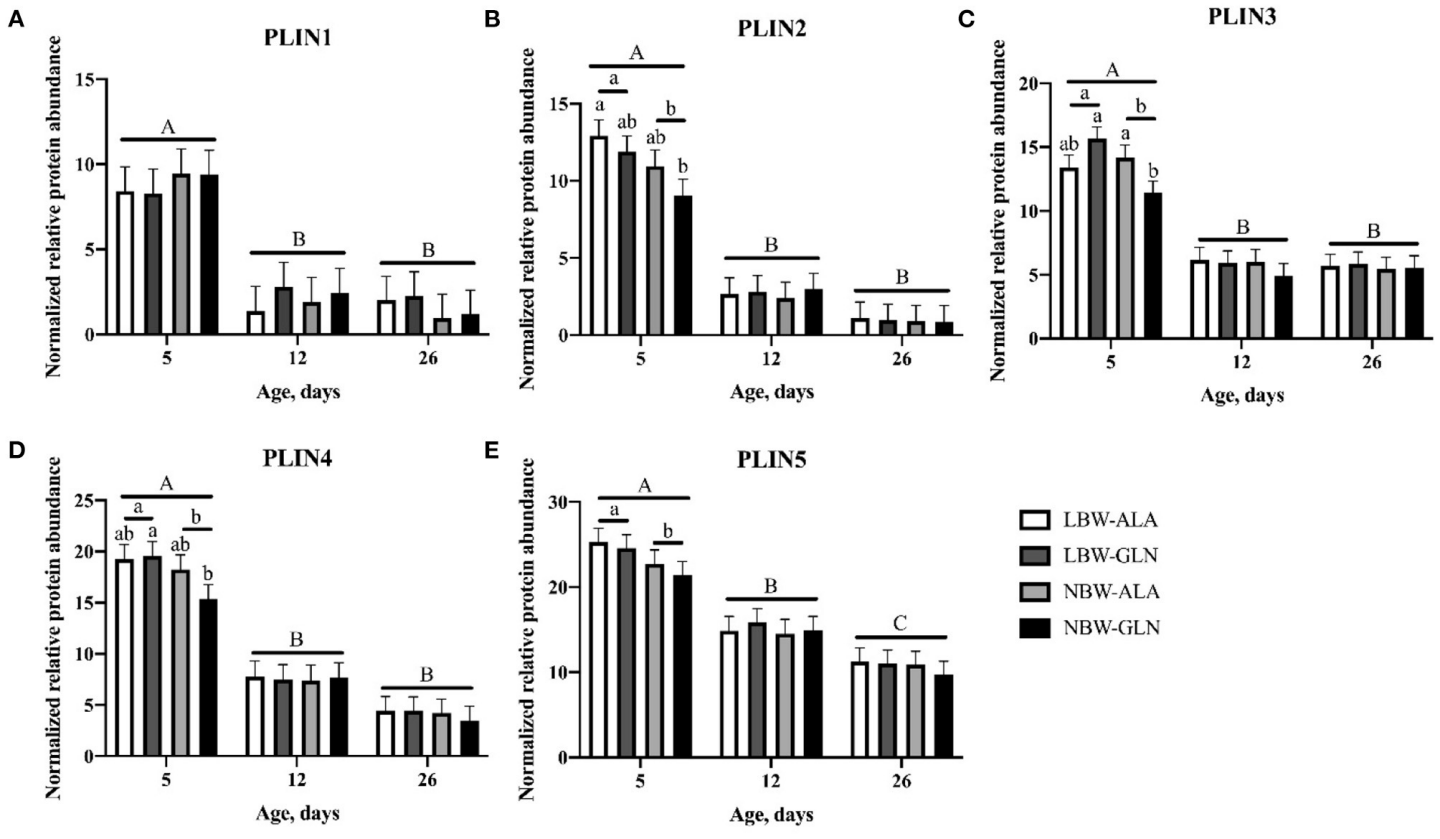
Normalized relative protein abundance of PLIN1 **(A)**, PLIN2 **(B)**, PLIN3 **(C)**, PLIN4 **(D)**, and PLIN5 **(E)** in *M. longissimus* of low- (LBW) and normal-birth-weight (NBW) piglets at 5, 12, and 26 dpn, supplemented with glutamine (GLN) or alanine (ALA) (*n* = 12 per group). Values represent LSmeans and SE in error bars of protein abundances normalized to total protein. ^a,b^Significant differences between piglets at the same age; ^A−*C*^Significant differences among ages (*p* < 0.05, Tukey–Kramer test).

Normalized protein abundances of PLIN1–5 within MLD were determined with western blots and are shown in Figure 5. A significant interaction effect among BiW, supplementation, and age was observed for the protein abundance of PLIN3 (*p* = 0.017), but for no other protein (*p* > 0.05). Age and BiW were the main factors influencing protein abundances of perilipins in MLD of piglets. The protein abundances of all five perilipins (Figures 5A–E) were higher in piglets at 5 dpn compared with animals at 12 dpn (*p* < 0.01) and 26 dpn (*p* < 0.01). The protein content of PLIN1 was not influenced by BiW, whereas PLIN3 and 4 protein abundances (Figures 5C,D) were higher in LBW-GLN compared with NBW-GLN piglets at 5 dpn (*p* < 0.001 and *p* = 0.021, respectively). Regardless of supplementation, protein abundances of PLIN2–5 (Figures 5B–E) were higher in LBW piglets compared with their NBW littermates at 5 dpn (*p* = 0.015, *p* = 0.009, *p* = 0.012, and *p* = 0.016, respectively). However, an influence of Gln supplementation on protein abundances of PLIN1–5 was not observed (*p* = 0.642, *p* = 0.522, *p* = 0.578, *p* = 0.460, and *p* = 0.68, respectively).

### Correlations Among Traits

Since most investigated traits were age dependent, Spearman correlation coefficients were calculated for each age group separately. We detected relationships among investigated genes and between gene expression and phenotypic traits (Supplementary Table 1). The relative mRNA abundances of *PLIN2, 4*, and *5* were moderately or highly correlated among each other in all age groups (*r* > 0.50, *p* < 0.001). The *PLIN1* mRNA was moderately correlated with mRNA of *PLIN2, 4*, and *5* at 12 and 26 dpn (*r* > 0.6, *p* < 0.001). Furthermore, *ATGL* mRNA was closely correlated with *CGI-58* and *PLIN2, 4*, and *5* mRNA at 5 dpn (*r* > 0.4, *p* < 0.001), whereas *LPL* mRNA was correlated with *ATGL* and *CGI-58* only in piglets at 5 dpn (*r* > 0.6, *p* < 0.001). The protein abundances of the five investigated *PLIN*s were not as closely correlated as mRNA, with only weak-to-high correlation among PLIN2 and PLIN4 and 5 in all age groups (*r* > 0.3, *p* < 0.001). There was no clear relationship between gene expression and intramuscular adipocyte area, except a weak negative correlation between PLIN1, 4, and 5 and adipocyte area at 12 dpn (*r* < −0.3, *p* < 0.05). However, we observed weak or moderate correlations between intramyocelluar lipid droplet area and protein abundances of PLIN2–5 (*r* > 0.3, *p* < 0.05) at 12 dpn and mRNA of *PLIN1, 2, 4*, and *5* as well as protein abundance of PLIN2 and 5 at 26 dpn (*r* = 0.357, *p* < 0.05). The mean adipocyte diameter showed a low negative correlation with intramuscular adipocyte area (*r* = −0.355, *p* < 0.05) at 12 dpn and with *PLIN4* mRNA (*r* = −0.337, *p* < 0.05) at 26 dpn.

## Discussion

Our previous study indicated a clear difference between LBW and NBW piglets in the abundance of lipid droplets within muscle fibers at 5 dpn (5). The area percentage of intramyocellular lipid droplets decreased during the first days of life by more than 80%. The difference between LBW and NBW piglets disappeared with age. At the same time, intramuscular adipocytes started to develop with high individual variation. The current study was conducted to elucidate the process of lipid deposition and redistribution between muscle fibers and developing adipocytes as part of the muscle development and its modulation by Gln. Intramyocellular lipid droplets are vital organelles sequestering lipids, regulating intramyocelluar lipid homeostasis by preventing from lipotoxicity (11, 15). Within skeletal muscle, the perilipin family of proteins plays a vital role in lipid droplet metabolism (33). It is not known whether Gln can directly interact with and modulate perilipin family members, but PLINs are biomarkers for lipid deposition and adipocyte development within skeletal muscle in pigs (25, 26, 34). Therefore, we investigated the expression pattern of the five known members of the perilipin family and their related lipases in skeletal muscle of LBW piglets and their NBW littermates, which were orally supplemented with Gln or Ala during the first 12 days of life.

### Larger Intramuscular Adipocytes Were Observed in LBW Piglets

Myogenesis and adipogenesis are competitive processes during muscle development (7, 8). Thus, when the muscle growth is delayed, as reported in LBW piglets (5, 10), nutrients are available for the development of intramuscular adipocytes at the same time. Gondret et al. (10) observed a greater adipocyte diameter in the MST of LBW pigs at 112 kg compared with high birth weight pigs. In agreement with Gondret et al. (10), we observed larger adipocytes in the MST, but not MLD, of LBW compared with NBW piglets during the first weeks of life. In addition, LBW piglets had more large-size adipocytes in both muscles at 26 dpn, whereas NBW animals had more small-size adipocytes at 5 dpn. Together with the result of our previous study that LBW had more intramyocelluar lipid droplets compared with NBW piglets (5), this suggests that LBW piglets stored the lipids in intramyocellular lipid droplets rather than in adipocytes during the first days of life. Then, with increasing age, the lipids were transferred to intramuscular adipocytes. Overall, LBW piglets had more intramuscular lipid deposition during the suckling period than their NBW littermates.

### Different Localizations of Perilipins Within *M. longissimus* Suggest Diverse Functions

All five members of the perilipin family of proteins were detected in MLD of piglets in our study with mainly distinct localizations. The first member, PLIN1, was originally discovered in adipocytes (35) surrounding lipid droplets (36). Other PLINs were consecutively detected. The five members of the perilipin family are proteins associated with the surface of lipid droplets and function in the formation, stabilization, and utilization of lipid droplets (26). Wolins et al. (37) elucidated the process how PLINs mediate lipid droplet formation in cultured 3T3-L1 adipocytes. According to their studies, nascent lipid droplets are coated first by PLIN3 and 4, then PLIN2 is recruited to the surface of growing lipid droplets. Afterwards, PLIN1 starts to bind the mature lipid droplets, stabilizing and protecting them from lipolysis. However, PLIN1 starts to promote triglyceride hydrolysis when it is phosphorylated by protein kinase A (38). In previous experiments, PLIN1 was discovered to be around large adipocytes (35, 39) and was thought to be restricted within white and brown adipose tissue or steroidogenic cells (40, 41). Our results indicate that PLIN1 can also be detected within skeletal muscle at the intramuscular adipocytes, in line with the findings of Gandolfi et al. (34). Furthermore, Skinner et al. (42) reported that PLIN1 can also be detected in the cytosol when it moves from smooth endoplasmic reticulum to mature adipocytes. While PLIN1 is a suitable marker for intramuscular fat deposition in pigs, PLIN2 was traditionally considered a marker for lipid droplets within skeletal muscle as it is located around small lipid droplets (34, 43). MacPherson et al., however, argued this point as the distribution of PLIN2 was only 60–80% parallel to intramuscular lipid droplets in some studies (44–46). Nevertheless, in most investigations, PLIN2 was found ubiquitously, appearing as the most abundant protein of the perilipin family in skeletal muscle (46, 47), which could not be concluded from our study. It shares a similar function and homology with PLIN1 and is able to replace PLIN1 when it is lacking (47, 48). The study of Xu et al. revealed that PLIN2 is competing with PLIN1 for binding at the surface of lipid droplets in adipocytes (49). There was no co-localization of PLIN1 and PLIN2 observed in MLD of piglets in our study. While PLIN1 and PLIN2 share similar functions, PLIN3 and PLIN4, both interacting with nascent lipid droplets, were localized at the periphery of muscle fibers and intramuscular adipocytes in our study. Previous studies have indicated that PLIN3 and 4 were also located cytosolically (21, 33, 50). However, in this study, we did not observe a clear association of PLIN3 and 4 with lipid droplets beside a weak cytosolic signal, which could indicate binding of smaller nascent lipid droplets that could not be detected under the microscope. The last member of this family, PLIN5, was localized within undefined cells between the muscle fibers in our study. It could not be clarified what kind of cells were stained and whether this was a specific binding of the PLIN5 antibody, although blocking the epitope with the respective peptide completely prevented antibody binding and staining. Previous studies found that PLIN5 could be recruited to the mitochondria to supply energy (33, 51); thus, it could be important during skeletal muscle growth. Altogether, the members of the perilipin family have different localizations within skeletal muscle tissue according to their distinct roles in lipid storage and metabolism.

### Differences Between LBW and NBW Piglets in Lipid Deposition at 5 dpn Were Associated With the Expression of *PLIN1–5* and Lipolysis-Related Genes

The expression of *PLIN*s and lipolysis-related genes was measured to elucidate whether the observed differences in intramuscular lipid deposition between LBW and NBW piglets are regulated by different *PLIN* family members and whether the availability of free Gln in the muscle was involved. There are many examples in the literature that *PLIN1* mRNA expression is correlated with fat deposition in different species, e.g., pigs (52), cattle (53), and humans (54). Our results indicated a higher *PLIN1* mRNA level, but not a higher protein level, in LBW piglets at 5 dpn compared with their NBW littermates. The lower protein abundance of PLIN1 in piglets at 26 dpn in comparison with animals at 5 dpn was in contrast to the increasing appearance of adipocytes with age (5). However, possible explanations for this expression pattern of PLIN1 are as follows: (i) the size of adipocytes is smaller in younger piglets; hence, more PLIN1 protein is required to enclose lipid droplets of small adipocytes as they have a larger summarized surface. (ii) The intramuscular fat was not evenly distributed in the muscle (5); thus, the results may vary within the same muscle, and different parts were used for histology and mRNA or protein extraction. (iii) The relative protein abundance was normalized to the total protein abundance, which is increasingly dominated by structural muscle proteins (55). This applies also for other PLIN proteins; thus, the age effect will not be discussed in the following.

PLIN2 was previously reported to stimulate uptake of fatty acids in transfected COS-7 cells (56) and promote lipid droplet enlargement in murine fibroblasts (57). Consistent with our published data of intramyocellular lipid droplets (5), we observed higher *PLIN2* mRNA and protein abundance in LBW compared with NBW piglets at 5 dpn in the current study. Thus, this variance of intramuscular lipid droplet content in piglets with different birth weight might result from the different expression pattern of *PLIN2*. Although, we observed weak-to-moderate positive correlations between intramyocellular lipid droplet area and PLIN2 protein abundance only at 12 and 26 dpn.

The protein abundance of PLIN3 and 4 was higher in LBW piglets at 5 dpn in comparison with NBW piglets, and both proteins had similar localizations in muscle tissue. We observed staining of small particles within particular muscle fibers, but different from the PLIN2 staining pattern. This is in accordance with previous studies indicating that PLIN3 functions in the biogenesis of small, nascent lipid droplets together with PLIN4 (21, 37, 58, 59). However, in PLIN3 blots, which were incubated with blocking peptide, the 47-kDa (theoretical size of PLIN3) band was only partially blocked, whereas a 63-kDa (similar to PLIN1) band was completely blocked. Therefore, the PLIN3 antibody possibly non-specifically binds to other members of the PLIN family. Additionally, the PLIN3 protein results should be considered with caution because of the very low mRNA abundance. Interestingly, PLIN4 blocking experiments indicated the size of PLIN4 protein at about 80 kDa (Supplementary Figure 3), which was smaller than the theoretical protein size (164 kDa). Whether it is an unknown isoform needs further clarification. Among the perilipin family, PLIN4 is the only protein that does not share the same PAT domain and a four-helix bundle with other PLINs and cannot mediate lipolysis (21, 26). Pourteymour et al. reported PLIN4 was more expressed in slow-twitch than in fast-twitch fibers in humans (60). Even though we did not investigate this specifically, it may also be similar in pigs, since most lipid droplets are located in slow-twitch fibers and are associated with PLINs.

Previous studies have suggested that PLIN5 is correlated with oxidative activities in skeletal muscle. Bosma et al. reported that overexpression of PLIN5 in the tibialis anterior muscle of rats increased oxidative gene expression and intramyocellular lipid content (61). Furthermore, this group reported higher interactivity of lipid droplets and mitochondria in human vastus lateralis muscle, rat tibialis anterior muscle, or cultured HEK293 cells upon overexpression of *PLIN5* (62). In our study, LBW piglets had a higher level of *PLIN5* mRNA and protein than NBW piglets at 5 dpn, regardless of supplementation. This suggests more oxidative activities in skeletal muscle of LBW piglets in the first days of life, probably to generate energy for muscle growth. Furthermore, PLIN5 protein was negatively correlated with adipocyte diameter in piglets at 26 dpn, suggesting PLIN5 is not involved in adipocyte growth and utilization of the lipids stored in intramyocellular lipid droplets.

Altogether, PLIN2–5 were more abundant in muscle tissue of LBW piglets than in NBW animals during their first days of life. A possible explanation for this observation may be that LBW piglets expend fewer fatty acids for their muscle and body growth than their NBW littermates, and the excess fatty acids are synthesized to triglycerides and stored in intramyocellular lipid droplets, preventing lipotoxicity. However, with increasing age, body growth or lipid deposition in adipocytes requires more energy, and the excess lipids in lipid droplets are then utilized. There is no indication from the current study that Gln supplementation alters the expression level of PLINs in porcine skeletal muscle.

The uptake of fatty acids from the blood is an important energy source for skeletal muscle metabolism, and LPL plays an important role in triacylglycerol hydrolysis from lipoproteins (63, 64). It is a rate-limiting lipase during this process and is mainly synthesized in skeletal muscle (64, 65). In a previous study, a tissue-specific over-expression of *LPL* was reported to increase the fatty acid content in mouse skeletal muscle (66, 67). Therefore, in the current study, we hypothesized that *LPL* expression was positively related to intramuscular lipid droplets, which are synthesized *de novo* from fatty acids (16). In the present study, relative *LPL* mRNA expression was significantly higher in piglets at 26 dpn compared with younger animals at 5 and 12 dpn, indicating an increasing demand of fatty acids in porcine skeletal muscle during the early postnatal period. The mRNA level of *LPL* was not influenced by BiW, suggesting that the higher content of intramyocellular lipid droplets in LBW piglets may not result from increased fatty acid uptake.

In addition to promoting lipid droplet formation and stabilization, PLINs were reported to modulate hydrolysis of the main content of lipid droplets, the triglycerides, together with ATGL and its coactivator CGI-58 (44). Moreover, ATGL is a vital lipase in skeletal muscle (41). During lipolysis within adipocytes, PLIN1 is phosphorylated first, then CGI-58 combined with PLIN1 is released from the surface of lipid droplets and activates ATGL (33). Subsequently, ATGL interacts with triacylglycerols of the lipid droplet cores and metabolizes them to diacylglycerols and fatty acids (33). In non-adipose tissue, e.g., myocytes, where PLIN1 is lacking, ATGL and CGI-58 could possibly mediate lipolysis with PLIN2 without being phosphorylated (44, 68, 69). Perilipin 3 and 5 could be both phosphorylated with stimulation to promote lipolysis in skeletal muscle (69). However, few studies have investigated the interaction of PLIN3 and lipases. Additionally, PLIN5 is able to bind CGI-58 or ATGL separately and is the only member of the PLIN family that binds ATGL directly (41, 70, 71). However, the interaction of PLIN5 and ATGL prevents lipolysis unless phosphorylation of PLIN5 (41). Our results indicated that mRNA expression of *CGI-58* was not influenced by BiW or Gln supplementation despite the difference in lipid droplet distribution between LBW and NBW piglets at 5 dpn. Moreover, *CGI-58*, as a regulator of ATGL activity (72), exhibited higher mRNA expression in piglets at 12 and 26 dpn in comparison with piglets at 5 dpn, indicating increased lipolysis activity with increasing age. However, the lipolysis rate of triglycerides in skeletal muscle is controlled by ATGL (69), but its relative mRNA expression in these animals was not influenced by BiW, supplementation, or age. Whether post translationally the regulation of ATGL played a role needs further clarification.

### PLINs Were Closely Correlated to Lipases Within *M. longissimus*

Relative mRNA expressions of *PLIN2, 4*, and *5* were closely correlated among each other within skeletal muscle of piglets in this study, suggesting their similar or interactive roles in intramuscular lipid droplet mobilization during the first weeks of life. Moreover, although *LPL* was not influenced by BiW, supplementation, or age in this study, its mRNA expression showed moderate correlations with the mRNA abundance of *PLIN2, PLIN5, ATGL*, and *CGI-58* in piglets at 5 dpn. This could be a sign of LPL's function in intramuscular lipid droplet formation together with these genes. In addition, as ATGL-mediated lipolysis requires interactions with PLIN family members (41), close correlations among *ATGL* mRNA and *PLIN*s in animals at 5 and 26 dpn were observed in this study. Consistent with that, the study of MacPherson et al. (44, 69) revealed distinct roles of PLINs in mediating lipolysis. Overexpression of *PLIN5* correlated with an increase of *ATGL* expression to maintain lipid homeostasis in the study of Bosma et al. (61). All these data revealed that *PLIN* family members are closely correlated with the important lipases in skeletal muscle.

## Conclusions

Perilipins have their distinct roles in regulating the lipid droplet formation, distribution, and mobilization in porcine skeletal muscle. Their expression and function are closely related as indicated by high correlations among *PLIN2*–*5* at both mRNA and protein level. The results revealed BiW as an important factor influencing their expression pattern, with a higher expression of most *PLIN*s in LBW piglets at 5 dpn. Furthermore, the close interactive correlations among *PLIN* family and related lipases suggested the *PLIN*s play important roles in lipolysis together with *LPL, ATGL*, and *CGI-58* within porcine skeletal muscle in the early postnatal phase. Oral supplementation with Gln had only negligible effects on the expression level of *PLIN*s and related lipases.

## Data Availability Statement

The raw data supporting the conclusions of this article will be made available by the authors, without undue reservation.

## Ethics Statement

The animal study was reviewed and approved by State Office for Agriculture, Food Safety and Fishing Mecklenburg Western Pomerania, Germany (permission No. 7221.3-1-026/16).

## Author Contributions

YZ performed most experiments, analyzed the data, and drafted the manuscript. EA designed the study, performed parts of the experiments and analyses, and helped with writing the manuscript. CM designed and supervised the animal experiment, provided resources, acquired funding, and revised the manuscript. MS, ZL, and JS performed the animal experiment, provided resources, and reviewed and edited the manuscript. SM supervised and administered the project, provided resources, and reviewed and edited the manuscript. All authors approved the final submitted manuscript.

## Conflict of Interest

The authors declare that the research was conducted in the absence of any commercial or financial relationships that could be construed as a potential conflict of interest.
