# Supplementary material for: Distinct Roles of Perilipins in the Intramuscular Deposition of Lipids in Glutamine-Supplemented, Low-, and Normal-Birth-Weight Piglets
Source: Front Vet Sci. 2021 Jun 21;8:633898. doi: 10.3389/fvets.2021.633898 (PMC8257002; doi:10.3389/fvets.2021.633898)
Supplement: Supplementary file 1 [file Data_Sheet_1.pdf]

Supplementary Material

Supplementary Table 1. Spearman correlation coefficients among mRNA and protein abundances of PLIN family members and related lipases and histological traits of lipid deposition within M. longissimus in piglets at 5, 12 and 26 dpn.

**Age**									**5 dpn**							
	**Items**	**PLIN1 mRNA**	**PLIN2 mRNA**	**PLIN4 mRNA**	**PLIN5 mRNA**	**LPL mRNA**	**ATGL mRNA**	**CGI-58 mRNA**	**PLIN1 protein**	**PLIN2 protein**	**PLIN3 protein**	**PLIN4 protein**	**PLIN5 protein**	**LDs area**	**Adipo area**	**MAD**
	PLIN1 mRNA		0.228	0.267	0.077	**0.356**	0.044	0.171	0.012	0.000	0.114	0.139	-0.065	0.183	0.103	-0.018
	p-value		0.119	0.067	0.603	0.013	0.765	0.245	0.935	0.998	0.442	0.345	0.662	0.212	0.487	0.903
	PLIN2 mRNA	**0.778**		**0.511**	**0.559**	**0.540**	**0.575**	**0.598**	-0.259	0.110	0.052	**0.376**	-0.243	0.231	0.051	-0.114
	p-value	<.0001		0.000	<.0001	<.0001	<.0001	<.0001	0.075	0.457	0.727	0.009	0.096	0.115	0.728	0.441
	PLIN4 mRNA	**0.735**	**0.864**		**0.798**	**0.465**	**0.543**	**0.524**	-0.221	0.115	0.270	0.177	0.048	0.179	0.055	0.022
	p-value	<.0001	<.0001		<.0001	0.001	<.0001	0.000	0.132	0.435	0.064	0.230	0.746	0.223	0.711	0.884
	PLIN5 mRNA	**0.778**	**0.857**	**0.883**		0.384	**0.438**	**0.422**	-0.136	0.280	0.241	**0.388**	0.024	0.080	0.038	0.055
	p-value	<.0001	<.0001	<.0001		0.007	0.002	0.003	0.355	0.054	0.099	0.007	0.873	0.590	0.797	0.712
	LPL mRNA	**0.311**	**0.294**	0.205	0.223		**0.639**	**0.643**	-0.214	-0.230	0.020	0.031	**-0.326**	0.016	0.186	-0.098
	p-value	0.032	0.043	0.161	0.127		<.0001	<.0001	0.145	0.115	0.892	0.833	0.024	0.913	0.204	0.506
**12 dpn**	ATGL mRNA	-0.207	-0.113	-0.149	-0.205	0.267		**0.834**	**-0.303**	-0.119	0.250	0.121	**-0.339**	0.403	0.100	-0.064
	p-value	0.158	0.445	0.311	0.162	0.067		<.0001	0.036	0.421	0.086	0.411	0.018	0.005	0.497	0.665
	CGI-58 mRNA	**0.416**	**0.487**	**0.407**	**0.325**	**0.547**	0.100		**-0.415**	-0.124	0.182	0.144	**-0.393**	0.305	0.082	-0.045
	p-value	0.003	0.000	0.004	0.024	<.0001	0.498		0.003	0.402	0.215	0.329	0.006	0.035	0.578	0.761
	PLIN1 protein	0.038	0.052	0.073	0.166	0.284	-0.154	0.000		-0.101	-0.087	0.004	-0.034	0.166	-0.010	-0.205
	p-value	0.799	0.725	0.620	0.260	0.050	0.297	0.999		0.496	0.558	0.980	0.818	0.260	0.944	0.162
	PLIN2 protein	0.030	0.070	0.160	0.135	0.172	0.171	0.146	0.324		0.110	**0.546**	**0.376**	-0.067	-0.101	0.276
	p-value	0.839	0.635	0.278	0.359	0.242	0.246	0.324	0.025		0.455	<.0001	0.009	0.649	0.493	0.057
	PLIN3 protein	0.167	0.265	0.258	**0.325**	0.129	0.104	0.021	0.195	0.259		0.051	-0.029	0.258	-0.040	0.048
	p-value	0.258	0.068	0.077	0.024	0.384	0.480	0.890	0.185	0.075		0.731	0.843	0.076	0.788	0.744

	PLIN4 protein	0.119	0.136	0.147	0.167	**0.341**	0.243	0.174	**0.431**	**0.663**	0.221		0.143	0.129	0.030	0.151
	p-value	0.420	0.357	0.319	0.257	0.018	0.096	0.237	0.002	<.0001	0.130		0.332	0.383	0.842	0.306
	PLIN5 protein	-0.223	-0.085	-0.109	-0.118	0.026	**0.358**	-0.055	0.172	**0.428**	0.166	**0.564**		**-0.368**	-0.061	**0.358**
	p-value	0.127	0.567	0.459	0.423	0.860	0.013	0.709	0.242	0.002	0.260	<.0001		0.010	0.679	0.012
	LDs area	-0.090	-0.141	-0.112	-0.062	-0.063	**0.420**	-0.143	0.114	**0.500**	0.309	**0.452**	**0.388**		-0.012	-0.078
	p-value	0.545	0.340	0.449	0.678	0.669	0.003	0.333	0.439	0.000	0.032	0.001	0.007		0.938	0.600
	Adipo area	**-0.350**	**-0.298**	**-0.301**	**-0.325**	-0.136	**0.396**	-0.208	0.127	-0.111	-0.068	0.079	0.222	0.271		0.284
	p-value	0.015	0.039	0.038	0.024	0.356	0.005	0.156	0.389	0.452	0.647	0.591	0.130	0.063		0.051
	MAD	0.016	0.033	0.033	-0.074	-0.016	-0.141	-0.025	0.053	-0.134	-0.229	0.104	0.191	**-0.355**	0.067	
	p-value	0.915	0.822	0.823	0.617	0.913	0.340	0.866	0.723	0.364	0.118	0.480	0.194	0.013	0.650	
	PLIN1 mRNA															
	p-value															
	PLIN2 mRNA	**0.625**														
	p-value	<.0001														
	PLIN4 mRNA	**0.621**	**0.521**													
	p-value	<.0001	0.000													
	PLIN5 mRNA	**0.649**	**0.668**	0.797												
	p-value	<.0001	<.0001	<.0001												
**26 dpn**	LPL mRNA	**0.372**	**0.286**	0.204	**0.307**											
	p-value	0.009	0.049	0.165	0.034											
	ATGL mRNA	0.075	**0.378**	0.128	0.129	0.221										
	p-value	0.613	0.008	0.385	0.384	0.132										
	CGI-58 mRNA	0.179	**0.328**	0.231	0.255	**0.300**	**0.577**									
	p-value	0.223	0.023	0.114	0.080	0.038	<.0001									
	PLIN1 protein	0.097	0.120	0.076	0.047	0.237	**0.292**	0.056								
	p-value	0.510	0.415	0.606	0.751	0.105	0.044	0.705								
	PLIN2 protein	0.118	**0.295**	0.246	0.249	0.105	0.103	0.031	**0.409**							
	p-value	0.426	0.042	0.092	0.088	0.478	0.488	0.834	0.004							

PLIN3 protein	**-0.369**	-0.176	0.026	-0.229	-0.211	**0.289**	0.075	0.152	0.276					
p-value	0.010	0.230	0.859	0.117	0.151	0.047	0.614	0.303	0.058					
PLIN4 protein	0.164	0.280	0.129	0.046	0.123	**0.305**	0.012	**0.633**	**0.617**	**0.392**				
p-value	0.264	0.054	0.381	0.757	0.405	0.035	0.938	<.0001	<.0001	0.006				
PLIN5 protein	**0.480**	**0.483**	**0.646**	**0.687**	0.110	0.010	0.025	0.217	**0.411**	-0.030	0.184			
p-value	0.001	0.001	<.0001	<.0001	0.455	0.947	0.864	0.139	0.004	0.841	0.211			
LDs area	**0.448**	**0.441**	**0.345**	**0.451**	0.272	0.265	0.149	0.200	0.422	0.023	**0.312**	**0.504**		
p-value	0.001	0.002	0.016	0.001	0.061	0.069	0.313	0.172	0.003	0.876	0.031	0.000		
Adipo area	**0.355**	**0.289**	0.120	0.182	**0.288**	-0.078	-0.071	0.311	0.195	-0.183	0.219	**0.306**	**0.427**	
p-value	0.013	0.047	0.416	0.215	0.047	0.599	0.631	0.031	0.185	0.214	0.136	0.034	0.003	
MAD	-0.100	-0.122	-0.337	-0.236	-0.174	-0.208	-0.283	0.126	-0.003	-0.082	0.139	-0.178	-0.088	0.263
p-value	0.498	0.409	0.019	0.107	0.237	0.156	0.052	0.394	0.986	0.582	0.346	0.227	0.551	0.071

LDs area: intramyocellular lipid droplets area; Adipo area: intramuscular adipocytes area; MAD: mean adipocyte diameter. All data was analyzed with CORR procedure of SAS.

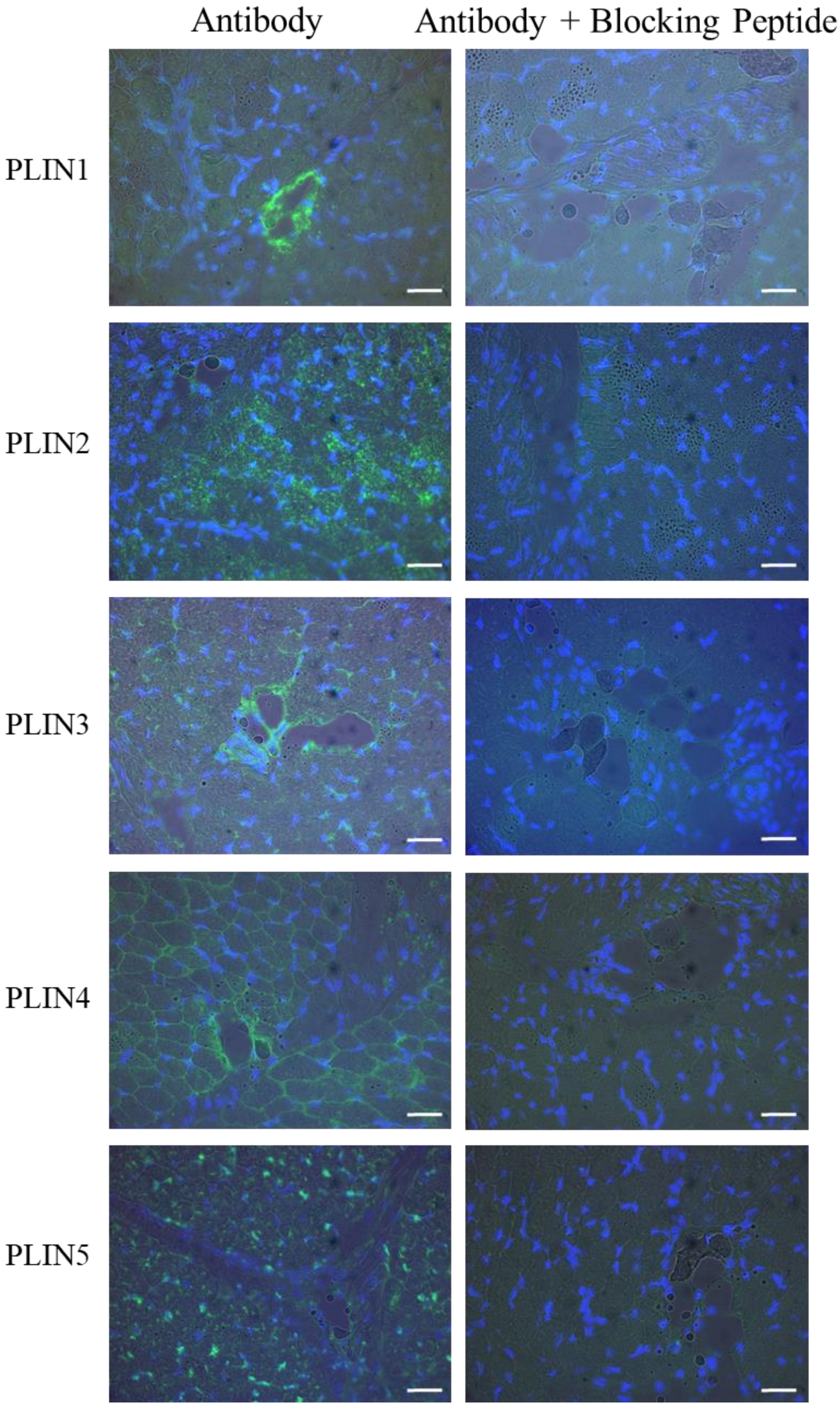
Antibody
Antibody + Blocking Peptide
PLIN1
PLIN2
PLIN3
PLIN4
PLIN5

Supplementary Figure 1. Muscle cross sections immunostained with antibodies for PLIN1-5 (green, left panel) or with antibodies preincubated with respective blocking peptides (right panel) in piglets at 12 dpn, respectively. All images are overlays of the fluorescence image (green) with nuclei staining (blue, Hoechst 33258) and bright field. Scale bars represent 50 µm.

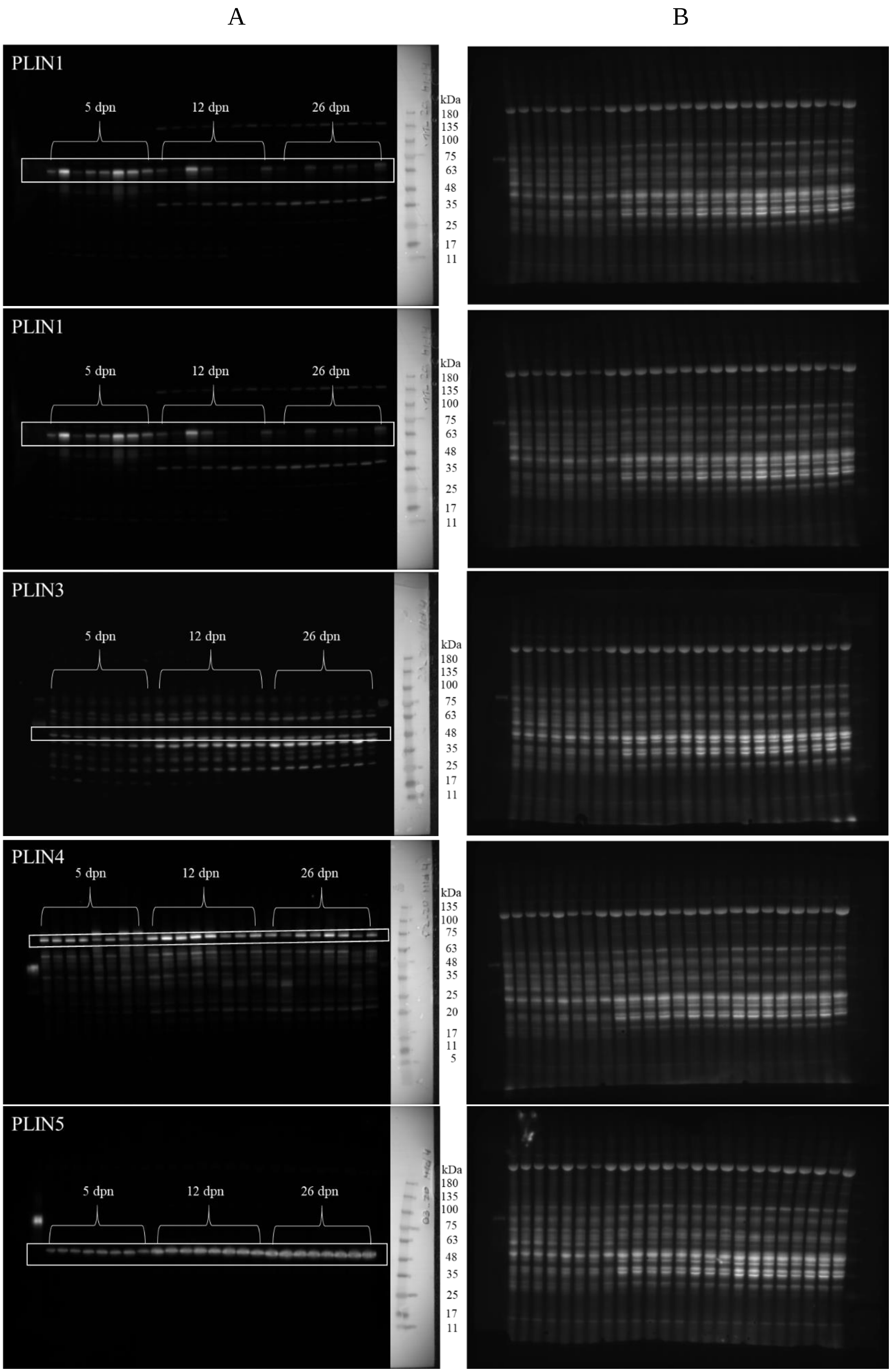
A
B
PLIN1
5 dpn
12 dpn
26 dpn
kDa
180
135
100
75
63
48
35
25
17
11
PLIN1
5 dpn
12 dpn
26 dpn
kDa
180
135
100
75
63
48
35
25
17
11
PLIN3
5 dpn
12 dpn
26 dpn
kDa
180
135
100
75
63
48
35
25
17
11
PLIN4
5 dpn
12 dpn
26 dpn
kDa
135
100
75
63
48
35
25
20
17
11
5
PLIN5
5 dpn
12 dpn
26 dpn
kDa
180
135
100
75
63
48
35
25
17
11

Supplementary Figure 2. Representative western blots for PLIN1-5 (left panel, A) and the total protein blots (right panel, B) used for normalization. Each lane contained 20 µg of muscle protein extract from M. longissimus of piglets at 5, 12 and 26 dpn, respectively. Target proteins are labelled in the white frame. Two animals from each group were included at different ages.

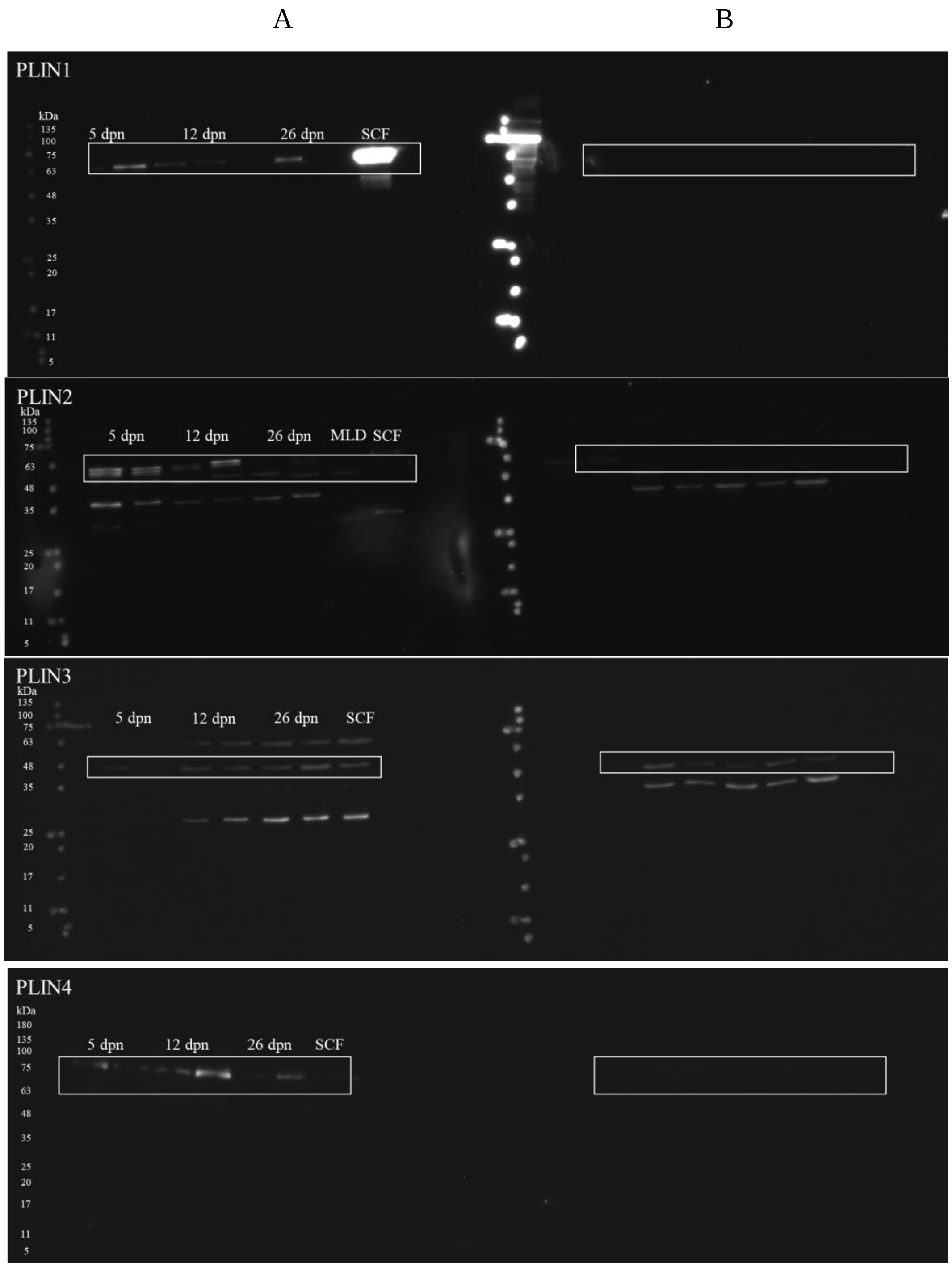
A
B
PLIN1
kDa
135
100
75
63
48
35
25
20
17
11
5
5 dpn
12 dpn
26 dpn
SCF
PLIN2
kDa
135
100
75
63
48
35
25
20
17
11
5
5 dpn
12 dpn
26 dpn
MLD
SCF
PLIN3
kDa
135
100
75
63
48
35
25
20
17
11
5
5 dpn
12 dpn
26 dpn
SCF
PLIN4
kDa
180
135
100
75
63
48
35
25
20
17
11
5
5 dpn
12 dpn
26 dpn
SCF

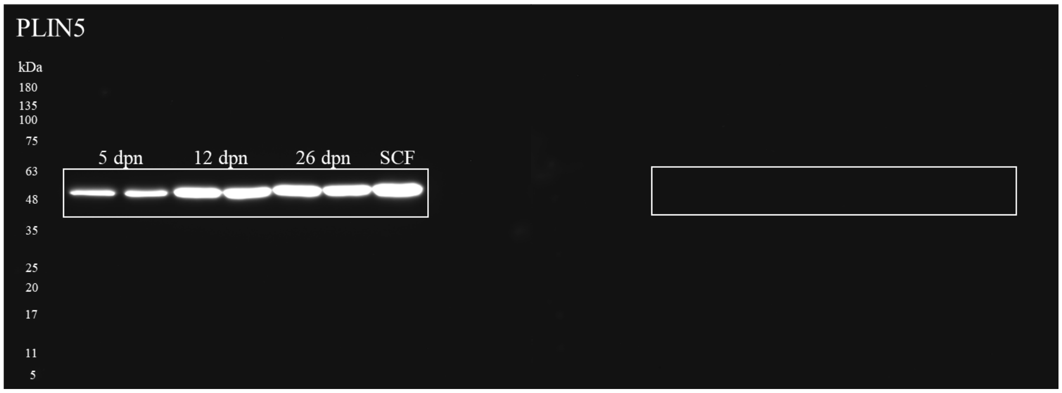

Supplementary Figure 3. Detection of non-specific binding of antibodies against perilipin family members PLIN1-5 in western blots. Blots were either incubated with the antibody (left panel) or with the antibody blocked with the respective blocking peptide in advance (right panel). Bands in the right panel (B) are non-specific. The specific bands (labelled in white frame) were blocked in panel B and were analysed for protein abundances of PLIN1-5. MLD: M. longissimus, SCF: subcutaneous fat.
